# Supplementary material for: International multicenter study comparing COVID-19 in patients with cancer to patients without cancer: Impact of risk factors and treatment modalities on survivorship
Source: eLife. 2023 Jan 30;12:e81127. doi: 10.7554/eLife.81127 (PMC9981148; doi:10.7554/eLife.81127)
Supplement: Supplementary file 1. [file elife-81127-supp1.docx]

Supplementary File 1. Comparing Mortality in Patients Treated with Remdesivir Alone or with Steroids for COVID-19.

| Patients groups | Outcome | Remdesivir and steroids | Remdesivir alone | *p-*value |
| --- | --- | --- | --- | --- |
| All patients | 30-Day death | 62/370 (16.8%) | 12/65 (18.5%) | 0.74 |
| Patients with Hypoxia | 30-Day death | 58/336 (17.3%) | 10/53 (18.9%) | 0.77 |
| With low-flow oxygen | 30-Day death | 3/43 (7%) | 0/8 (0%) | >.99 |
| With high-flow oxygen | 30-Day death | 5/18 (27.8%) | 6/19 (31.6%) | 0.8 |
| On ventilation at diagnosis | 30-Day death | 26/146 (17.8%) | 7/30 (23.3%) | 0.48 |

Note: The number of patients with data available for a variable is added as denominator in its analysis result.
