# Supplementary material for: International multicenter study comparing COVID-19 in patients with cancer to patients without cancer: Impact of risk factors and treatment modalities on survivorship
Source: eLife. 2023 Jan 30;12:e81127. doi: 10.7554/eLife.81127 (PMC9981148; doi:10.7554/eLife.81127)
Supplement: Supplementary file 2. [file elife-81127-supp2.docx]

Supplementary File 2. Timing of administration of corticosteroids as COVID-19 treatment and 30-day mortality

| Treatment and timing of initiation in relation to COVID-19 diagnosis | Alive | Dead | *p-*value |
| --- | --- | --- | --- |
|  | N/n (%) | N/n (%) |  |
|  |  |  |  |
| Corticos**teroids** |  |  |  |
| ≤ 3 days | 594/789 (75) | 131/165 (79) | 0.3 |
| > 3 days | 195/789 (25) | 34/165 (21) |  |
|  |  |  |  |
| ≤ 4 days | 633/789 (80) | 139/165 (84) | 0.2 |
| > 4 days | 156/789 (20) | 26/165 (16) |  |
|  |  |  |  |
| ≤ 5 days | 665/789 (84) | 147/165 (89) | 0.1 |
| > 5 days | 124/789 (16) | 18/165 (11) |  |
|  |  |  |  |
| ≤ 6 days | 700/789 (89) | 153/165 (93) | 0.1 |
| > 6 days | 89/789 (11) | 12/165 (7) |  |

Note:

1. Diagnosis of COVID-19 by PCR occurs around 2-3 days after onset of symptoms.

2. The number of patients with data available for a variable is added as denominator in its analysis result.
